# Yield and resource use efficiency of *Plukenetia volubilis* plants at two distinct growth stages as affected by irrigation and fertilization

**DOI:** 10.1038/s41598-017-18342-6

**Published:** 2018-01-08

**Authors:** He-De Gong, Yan-Jing Geng, Chun Yang, Dong-Ying Jiao, Liang Chen, Zhi-Quan Cai

**Affiliations:** 1 School of Geography, Southwest Forestry University, Kunming, 650224 China; 2 Key Laboratory of Tropical Plant Resources and Sustainable Use, Xishuangbanna Tropical Botanical Garden, Chinese Academy of Sciences, Mengla, 666303 China; 3 Hubei Key Laboratory of Edible Wild Plants Conservation and Utilization, Hubei Normal University, Huangshi, Hubei 435002 China

## Abstract

This study is to test how seedlings (vegetative) and large plants (reproductive) of an oilseed crop (*Plukenetia volubilis*) responded to regulated deficit irrigation techniques (conventional deficit irrigation, DI; alternative partial root-zone irrigation, APRI) in a tropical humid monsoon area. Seedlings were more sensitive to water deficit than large plants. Although APRI did better than DI in saving water for both seedlings and large plants at the same amount of irrigation, full irrigation (FI) is optimal for faster seedling growth at the expense of water-use efficiency (WUE). The seed number per unit area was responsible for the total seed oil yield, largely depending on the active process of carbon and nitrogen storages at the whole-plant level. The magnitude of the increase in total seed and seed oil yield by fertilization was similar under different irrigation regimes. Compared with FI, DI can save water, but reduced the total seed yield and had lower agronomic nutrient-use efficiency (NUE_agr_); whereas APRI had similar total seed yield and NUE_agr_, but reduced water use greatly. Although the dual goal of increasing the yield and saving water was not compatible, maintaining a high yield and NUEagr at the cost of WUE is recommended for *P. volubilis* plantation in t he water-rich areas.

## Introduction

Water stress is a major limitation for crop production in many areas of the world since it not only reduces cell growth rate, but also limits the crops’ reproductive process^1^. A major challenge in food production is to achieve the goal of increasing both food production and resource (mainly water and nitrogen) use efficiency^2,3^. To maintain sustainable production and efficient use of the limited water resources, various types of water-saving irrigation techniques have been widely introduced, many of them taking advantage of the fact that changes in hydraulic and chemical signals induced by rootzone drying caused partial closure of stomata and inhibition of leaf expansion^4,5^. It has been identified that regulated deficit irrigation (RDI) can save irrigated water up to 20–30% and increase water-use efficiency (WUE) greatly with a subtle or even positive impact on the yield and quality of some grain and fruit crops, especially in arid and semiarid regions^6,7^. Among the RDI techniques (alternate partial root-zone irrigation, APRI; conventional deficit irrigation, DI; subsurface irrigation, etc.), APRI has been found to be efficient in saving water and improving WUE while maintaining productivity in some agricultural and horticultural crops^8–10^. However, the adoption of such a water-saving practice is problematic in the tropical humid monsoon regions, where where annual rainfall is high and the wetting–drying cycles resulted in temporal changes in soil structure^10,11^. In addition, under natural conditions, soil moisture varies much less than the leaf-atmosphere flux, which fluctuates in response to a high frequency of atmospheric vapor pressure deficit (VPD). From a physiological perspective, VPD control has been hypothesized to play an important role in reducing the water flow rate and cumulative transpiration by suppressing the excess water driving force along the soil-plant-atmosphere continuum^1^. Exposure of crops to warmer and drier environments will increase leaf-air water VPD, resulting in increased drought susceptibility and reduced productivity, not only in arid regions but also in tropical monsoon regions with seasonal dry periods^12^. However, the high relative air humidity and low leaf-air water VPD during leaf expansion in the humid areas may hamper stomatal responsiveness to closing stimuli with a genotype-dependent effect^13^.

It still remains debatable if the water-saving techniques could achieve the dual goal of increasing crop yield and saving water, especially for the sparsely planted woody crops^14,15^. Moreover, to apply RDI effectively, one must predetermine the critical growth stage for a specific crop species and evaluate the relative sensitivity of crop plants to water deficit at various stages in their life cycle because larger plants use more water than smaller plants^16–18^. On the other hand, nutrient transport in the soil and absorption by roots are limited by water. Both biomass production and the yield of crops are co-limited by nutrient and water availabilities^19,20^. Normally, fertilization can raise grain yield and increase growers’ profits, but high application rates are not guaranteed to continually increase yield; instead, this can result in low nutrient use efficiency (NUE) and/or environmental issues^2^. If crop management technologies are properly used at a certain growth stage, a synergistic interaction between soil moisture and fertilizer on crop growth and yield may occur and can also increase WUE and NUE^2,3,21,22^.

*Plukenetia volubilis* Linneo, a tropical evergreen liana native to South America, is a promising new oilseed crop species belonging to the family Euphorbiaceae. *P. volubilis* plants grow continuously in tropical regions, and therefore flower and fruit almost continuously throughout the year. Each fruit is a capsule consisting of four-to-seven pods, with one seed per pod. The yield and quality of the seed oil of *P. volubilis* plants are highly variable and depend on environmental conditions and agricultural management practices^19,23–26^. Seed oil production of *P. volubilis* plants requires high amounts of fertilizer^25^; irrigation in the dry season is necessary for increasing the yield potential because *P. volubilis* plants grown under natural drought conditions have lower numbers of female flowers and higher fruit abortion compared to the well-watered plants^19^. Currently, the analysis of the impact of RDI and fertilization on the agronomic traits of *P. volubilis* plants has been limited. Therefore, the experiments for the current study were conducted to investigate the effects of two RDI approaches (i.e., APRI and DI) and fertilization on the plant physiology, growth, yield, and resource use efficiency of *P. volubilis* plants in southwest China, so as to provide a scientific basis for water and fertilization managements in the tropical humid monsoon regions. The objectives of this study are the following: (1) to evaluate the sensitiveness of plant growth in response to water stress between seedlings at the vegetative stage and large plants at the reproductive stage; (2) to determine if irrigation regimes could synergistically interact with fertilization rates to increase yield, WUE and NUE of *P. volubilis* plants in the field and (3) to determine the combination of RDI and fertilization to optimize seed oil yield and resource-use efficiency.

## Results

### Seedling experiment

Except for leaf N concentration, leaf and plant growth traits of *P. volubilis* were greatly influenced by regulated deficit irrigation (RDI) (Table 1). In general, the net photosynthetic rate (Pn), stomatal conductance (Gs,) transpiration rate (Tr), total biomass, leaf area index (LAI), specific leaf area (SLA) and photosynthetic nitrogen-use efficiency (PNUE) decreased, whereas root mass fraction (RMF), root/shoot (R/S) ratio and WUEi increased with decreasing amount of irrigation water. Total biomass was positively correlated to Pn and LAI (r = 0.60–0.94, *P* < 0.05, respectively) across all irrigation regimes, indicating that the decreased biomass was attributed to the reduced leaf photosynthetic rate and leaf area. The whole-plant water-use efficiency (WUEwp = seedling biomass increase per water applied) was highest in the two APRI regimes and was about onefold higher than that in full irrigation (FI). At the same amount of irrigation, total biomass, WUEi, and WUEwp values were higher in APRI than those in DI. Thus, compared to FI, APRI resulted in efficient water use, but impaired physiological parameters and decreased seedling growth. Across all irrigation regimes, Pn was positively correlated to Gs (r = 0.98, *P* < 0.01).

**Table 1:** Leaf physiological and the whole-plant traits in seedlings of *P. volubilis* plants under different irrigation treatments in the greenhouse.

Irrigation	Pn (μmolm^−2^s^−1^)	Gs (mmol m^−2^s^−1^)	Tr (μmolm^−2^s^−1^)	Leaf N conc. (g kg^−1^)	TB (g)	RMF (%)	R/S (%)	LAI	SLA (g cm^−2^)	PNUE (μmol m^−2^s^−1^g^−1^N)	WUEi (μmolmmol^−1^)	WUEwp (g kg^−1^H_2_O)
DI50	4.97d	0.14c	2.52c	29.1a	0.98d	15.22b	16.15b	0.39c	213.5b	17.08d	1.97ab	0.17b
APRI50	5.45c	0.15c	2.61bc	29.3a	1.44b	16.60a	16.81a	0.56b	210.1b	18.60c	2.09ab	0.37a
DI75	7.21b	0.26b	3.21b	28.4a	1.20c	13.26c	14.53c	0.46b	226.70ab	25.39b	2.24b	0.16b
APRI75	7.15b	0.25b	2.92b	28.2a	1.45b	13.77c	15.81b	0.67a	237.80b	25.35b	2.45a	0.38a
FI	8.11a	0.32a	4.43a	27.9a	1.51a	13.98c	14.12c	0.71a	245.6a	29.07a	1.83c	0.18b

Mean values (n = 5–6) within a column for each variable followed by different letters are significantly different at *P* < 0.05 level. DI, conventional deficit irrigation; APRI, alternative partial-root irrigation; FI, full irrigation. Pn, light-saturated photosynthetic rate; Gs, stomatal conductance; Tr, transpiration rate; TB, total biomass; RMF, root mass to plant mass fraction; R/S, root to shoot ratio; SLA, specific leaf area; LAI, leaf area index; PNUE, photosynthetic nitrogen-use efficiency; WUEi, instantaneous water-use efficiency; WUEwp, whole plant water-use efficiency.

### Field experiment

#### Leaf physiological and whole-plant traits

**Figure 1:**
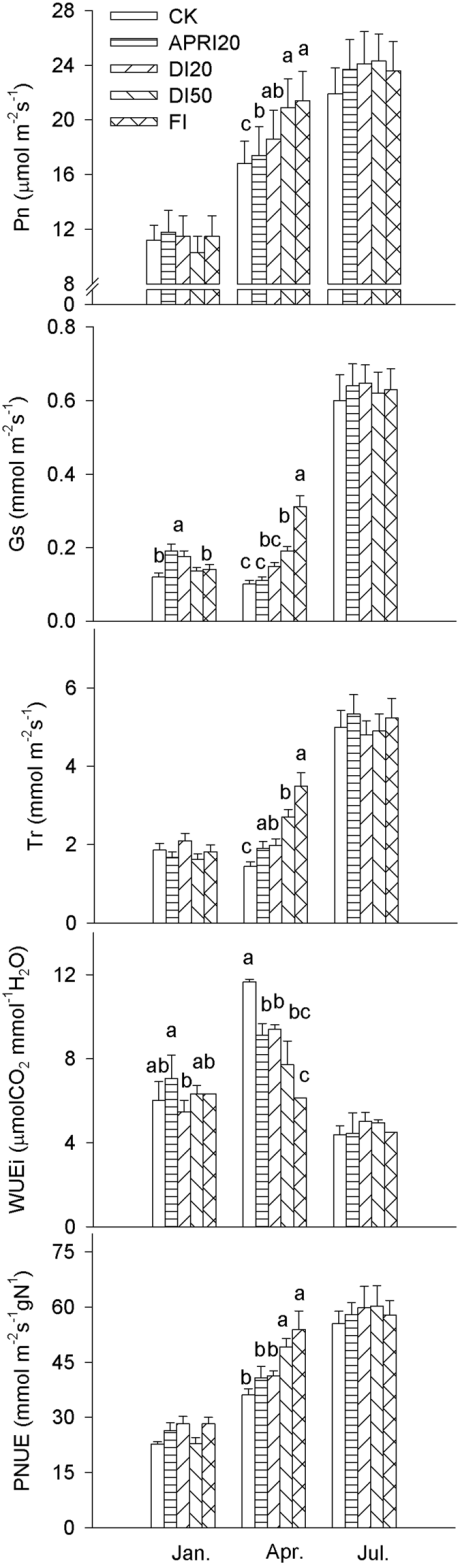
Effects of different irrigation treatments on the seasonal variations of leaf gas exchange parameters of *P. volubilis* plants under non-fertilized conditions in the field. The values (means ± SD, n = 5–6) with different letters within each season denote significantly at *P* < 0.05 level. ns, no significance; *P < 0.05; **P < 0.01; ***P < 0.001. CK, control = natural rainfed. Abbreviations of photosynthetic parameters and irrigation treatments are as defined in Table 1.

Pn, Gs, Tr and PNUE were highest in the wet season (July) and lowest in the cool and dry season (January); whereas the highest and the lowest WUEi was found in the hot and dry season (April) and the wet season, respectively, across all irrigation regimes (Fig. 1). Irrigation greatly influenced the gas exchange parameters in the hot and dry season, but this effect was minor in both the cool and dry season and the wet season. In the hot and dry season, Pn, Gs, Tr, and PNUE generally increased, whereas WUEi decreased with increasing amounts of the irrigation applied in the dry season; Pn was positively correlated with Gs (r = 0.88, *P* < 0.01), but PNUE was negatively correlated with WUEi across all irrigation regimes (r = −0.92, *P* < 0.01).

**Figure 2:**
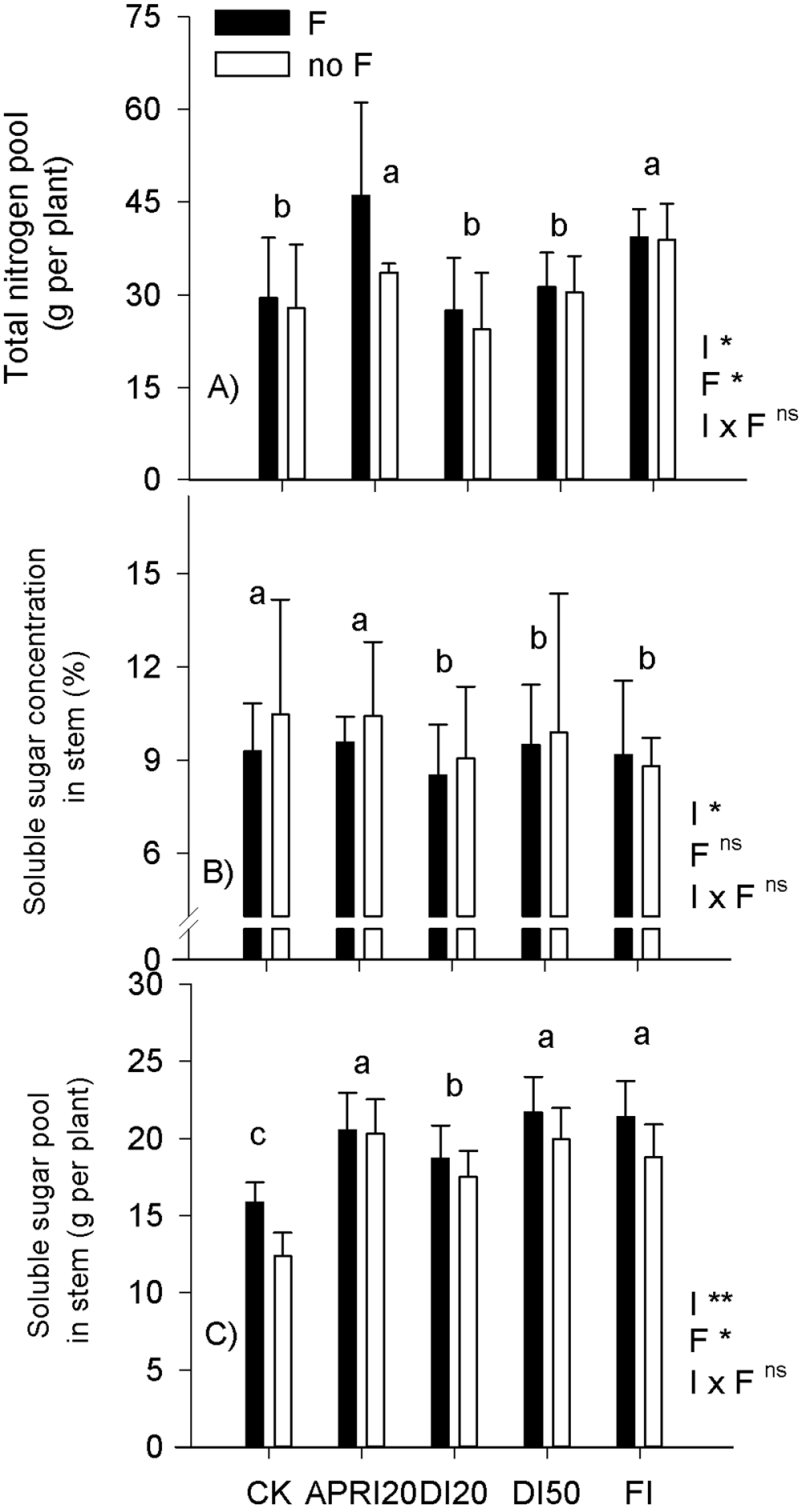
Effects of different irrigation (I) and fertilization (F) treatments on the total nitrogen pool, soluble sugar concentration and pool in stem of *P. volubilis* plants in the field. The values (means ± SD, n = 3–4) with different letters denote significantly at *P* < 0.05 level. ns, no significance; *P < 0.05; **P < 0.01. Abbreviations of irrigation treatments are as defined in Table 1.

There were no irrigation × fertilizer interactions on the whole-plant traits and nitrogen concentrations in the vegetative tissues of the field-grown *P. volubilis* plants (Table 2). *P. volubilis* plants allocated more biomass to stems and fruits (i.e., high SMF and HI), but much less to roots. Neither fertilization nor irrigation significantly affected the SMF and N concentrations of vegetative tissues. With decreasing amounts of the irrigation, SLA and LAI generally decreased; whereas fertilization increased total biomass, LMF, HI and LAI, but decreased R/S ratio (Table 2). At the same amount of irrigation (APRI20 vs. DI20), APRI had higher HI but lower total biomass than DI. There were no significant irrigation × fertilizer interactions on the total nitrogen pool of the vegetative tissues per plant, soluble sugar concentration and pool in stem, suggesting that C or N in response to irrigation was not significantly influenced by fertilizer level (Fig. 2). Fertilization increased total nitrogen pool and soluble sugar pool in stem, rather than the soluble sugar concentration in stem. Among the irrigation regimes, the highest values of total nitrogen pool and soluble sugar pool in stem were found in APRI20 and FI, and in APRI20, DI50 and FI, respectively.

**Table 2:** The whole-plant traits and N concentrations in different organs of *P. volubilis* plants under different irrigation (I) and fertilization (F) treatments in the field.

Treatments	TB (kg)	RMF (%)	SMF (%)	LMF (%)	HI (%)	R/S (%)	SLA (g cm^−2^)	LAI	Leaf N conc. (g kg^−1^)	Stem N conc. (g kg^−1^)	Root N conc. (g kg^−1^)
CK	4.21ab	3.46c	40.67	9.53c	35.21c	8.43c	152.34c	1.54bc	29.66	8.30	12.83
APRI20	3.06b	4.77b	49.66	10.77bc	38.90a	9.59b	160.75bc	1.32c	29.33	8.09	13.10
DI20	4.50a	4.53b	50.06	8.69d	35.44c	9.08c	168.82b	1.48c	30.81	8.85	15.16
DI50	3.50b	5.09a	58.06	11.75b	36.72bc	8.74c	167.63b	1.62b	30.01	8.24	14.14
FI	4.73a	5.96a	44.63	13.46a	37.11b	13.46a	180.13a	2.74a	30.53	8.16	13.46
*Means*	*4.00*	*4.76*	*48.62*	*10.84*	*36.68*	*9.86*	*165.93*	*1.74*	*30.07*	*8.33*	*13.74*
CK + F	4.50b	3.23c	46.78	10.56bc	36.45b	6.98c	168.60b	2.00bc	29.28	7.22	14.35
APRI20 + F	4.32b	4.77b	47.24	9.74c	42.36a	9.80b	176.71b	1.87c	25.95	7.60	13.89
DI20 + F	5.07a	5.99a	47.13	11.77a	35.12b	12.73a	157.73c	2.28b	29.96	9.75	15.29
DI50 + F	3.89b	3.96c	58.50	9.73c	36.53b	6.69c	180.77a	1.66c	27.02	8.79	13.01
FI + F	4.45ab	4.72b	53.42	13.48a	40.11a	8.80b	187.10a	2.67a	28.59	9.87	14.45
*Means*	*4.45*	*4.53*	*50.61*	*11.06*	*38.11*	*9.00*	*174.18*	*2.10*	*28.16*	*8.65*	*14.20*
Significant											
I	*	*	ns	*	*	**	*	*	ns	ns	ns
F	*	ns	ns	*	*	ns	ns	**	ns	ns	ns
I × F	ns	ns	ns	ns	ns	ns	ns	ns	ns	ns	ns

The values (means, n = 4–5) with different letters each variable denote significantly at *P* < 0.05 level at the same fertilized conditions. ns, no significance; *P < 0.05; **P < 0.01; ***P < 0.001. LMF, leaf mass to plant mass fraction; HI, harvest index. Abbreviations of growth parameters and irrigation treatments are as defined in Table 1.

#### Yield and resource use efficiency

**Figure 3:**
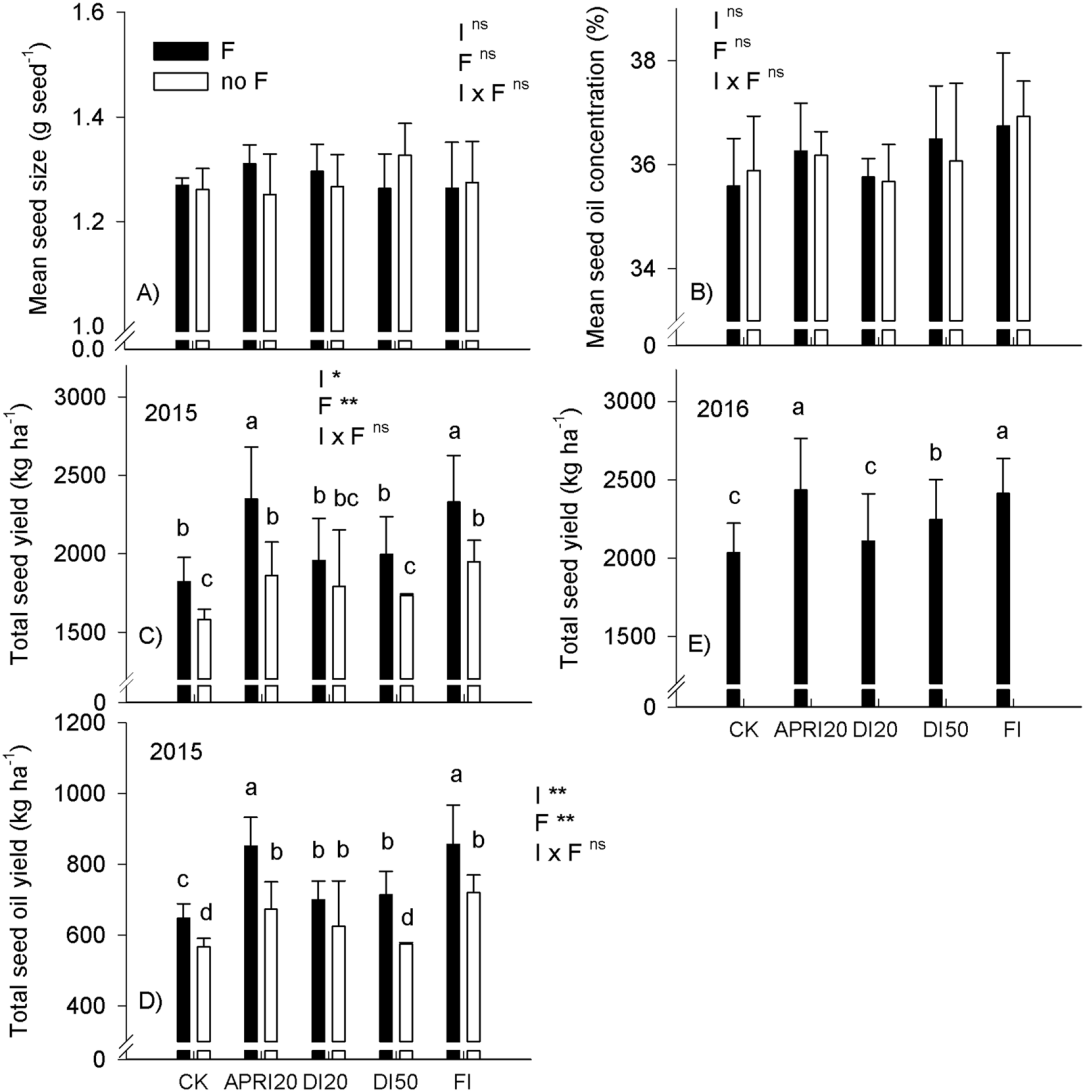
Effects of different irrigation (I) and fertilization (F) treatments on the yield components and total seed or seed oil yield over the growing season of *P. volubilis* plants in the field. The values (means ± SD, n = 3–6) with different letters denote significantly at *P* < 0.05 level. ns, no significance; *P < 0.05; **P < 0.01. Abbreviations of irrigation treatments are as defined in Table 1.

Neither irrigation nor fertilization influenced the dynamic pattern of the fruit ripening of *P. volubilis* plants (Table 3). The relatively larger seed size and higher seed oil concentration were observed in March, whereas higher seed yield was found in April across different sampling dates. Mean seed size and mean seed oil concentration over the growing season in 2015 were not influenced by irrigation or fertilization (Fig. 3A,B). However, ranging from 1581.4 to 2348.3 kg ha^−1^ and from 567.6 to 856.0 kg ha^−1^, respectively, total seed yield and total seed oil yield at the sub-plot level among the different treatments were significantly affected by irrigation and fertilization, rather than by the irrigation × fertilization interactions (Fig. 3C,D). Thus, the magnitude of increase in total seed and seed oil yield by fertilization was similar under different irrigation regimes. With the lowest values occurring in the rainfed (control), total seed and seed oil yield generally increased with increasing amount of the irrigation in DI regimes, and the highest values were found in APRI20 and FI when combined with fertilized conditions. Seed yields, ranging from 2034.5 to 2434.6 kg ha^−1^ under fertilized condition, were higher in 2016 than those in 2015 (Fig. 3E). Non-additive effect of irrigation on total seed yield was found between two years, as there was no year × irrigation interaction (F = 1.58, *P* > 0.05).

**Table 3:** Seasonal dynamic of the seed size, seed oil concentration and seed yield of *P. volubilis* plants under different irrigation (I) and fertilization (F) treatments in the field. ns, no significance; *P < 0.05; **P < 0.01.

Treatments	Seed size (g per seed)						Seed oil concentration (%)					Seed yield (kg ha^−1^)			
	Dec.	Jan.	Feb.	Mar.	Apr.	Dec.	Jan.	Feb.	Mar.	Apr.	Dec.	Jan.	Feb.	Mar.	Apr.
CK	1.30	1.25	1.21	1.30	1.25	36.85	35.88	34.14	36.22	36.36	423.1	214.0	146.3	93.5	704.1
APRI20	1.30	1.18	1.27	1.35	1.17	35.48	35.97	36.45	36.4	36.6	485.3	322.5	129.2	342.6	580.2
DI20	1.36	1.22	1.29	1.24	1.22	35.76	34.96	36.75	35.08	35.81	404.6	309.6	235.2	312.4	531.3
DI50	1.27	1.32	1.3	1.43	1.32	35.83	37.04	35.18	38.05	34.28	426.4	179.1	169.0	344.0	615.3
FI	1.32	1.21	1.25	1.39	1.21	36.64	35.9	37.67	37.26	37.15	431.5	387.1	214.8	308.6	607.2
CK + F	1.27	1.26	1.27	1.29	1.26	36.01	34.54	34.79	36.77	35.84	385.9	310.3	196.4	324.9	603.3
APRI20 + F	1.34	1.28	1.31	1.35	1.27	36.1	36.27	37.06	37.06	34.8	628.6	527.9	186.3	439.3	566.6
DI20 + F	1.22	1.3	1.31	1.36	1.29	36.17	35.41	35.92	35.94	35.37	486.7	412.3	183.9	298.5	575.3
DI50 + F	1.37	1.21	1.24	1.29	1.21	37.14	35.6	36.95	37.56	35.22	422.2	322.9	245.2	323.7	681.5
FI + F	1.34	1.18	1.24	1.37	1.18	35.13	38.37	36.06	38.05	36.11	438.8	389.6	264.6	467.2	768.8
*Means*	1.309	1.241	1.27	1.34	1.238	36.111	35.10	36.10	36.84	35.75	453.31	337.53	197.09	325.5	623.4
Significant level															
I	ns	ns	ns	ns	ns	ns	ns	ns	ns	ns	*	*	**	*	*
F	ns	ns	ns	ns	ns	ns	ns	ns	ns	ns	*	**	**	**	*
I** × **F	ns	ns	ns	ns	ns	ns	ns	ns	ns	ns	ns	*	ns	ns	*

Abbreviations of irrigation treatments are as defined in Table 1.

**Figure 4:**
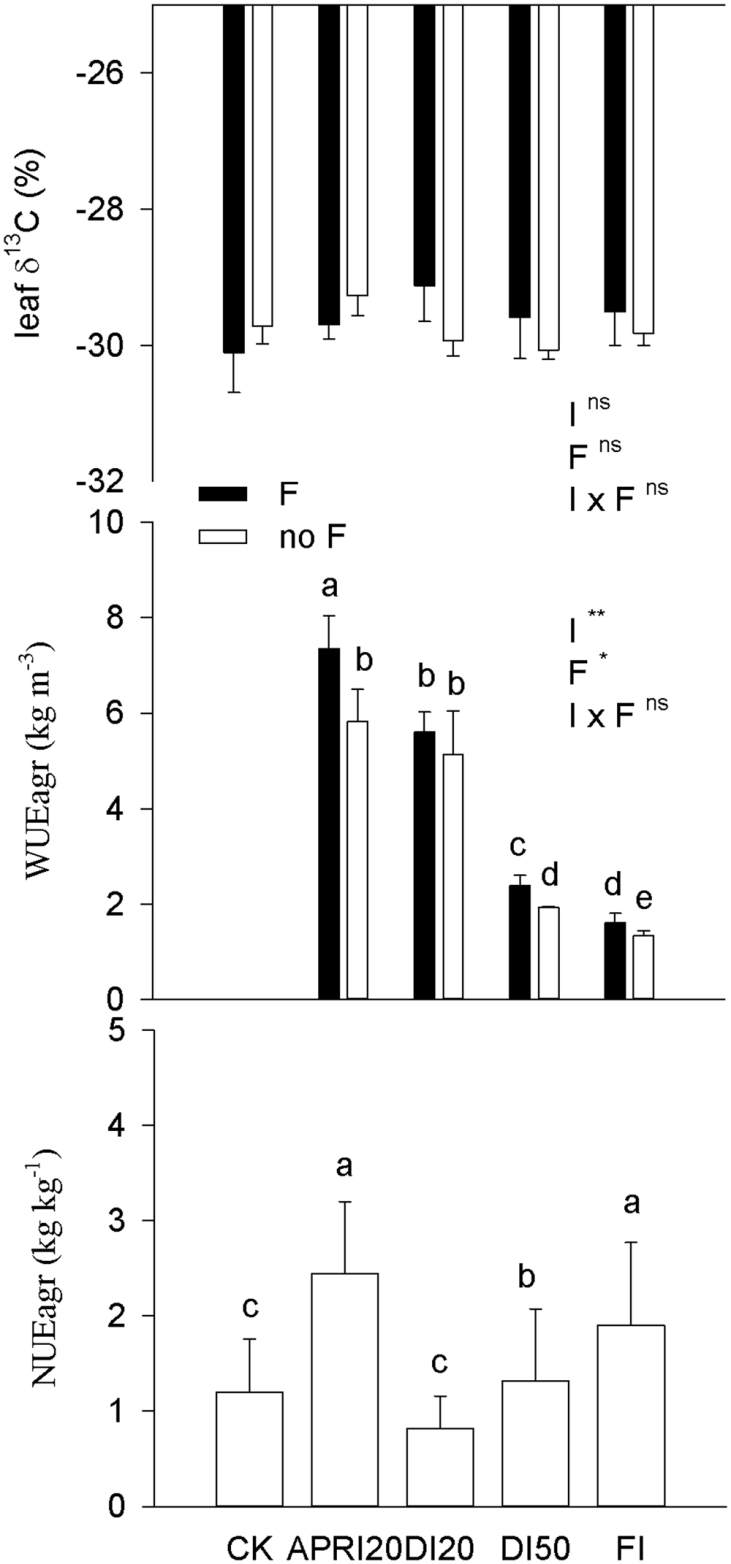
Effects of different irrigation (I) and fertilization (F) treatments on the leaf δ^13^C value, agronomic water-use efficiency (WUEagr) and agronomic nutrient-use efficiency (NUEagr) of *P. volubilis* plants in the field. The values (means ± SD, n = 3–4) with different letters denote significantly at *P* < 0.05 level. ns, no significance; *P < 0.05; **P < 0.01. Abbreviations of irrigation treatments are as defined in Table 1.

**Figure 5:**
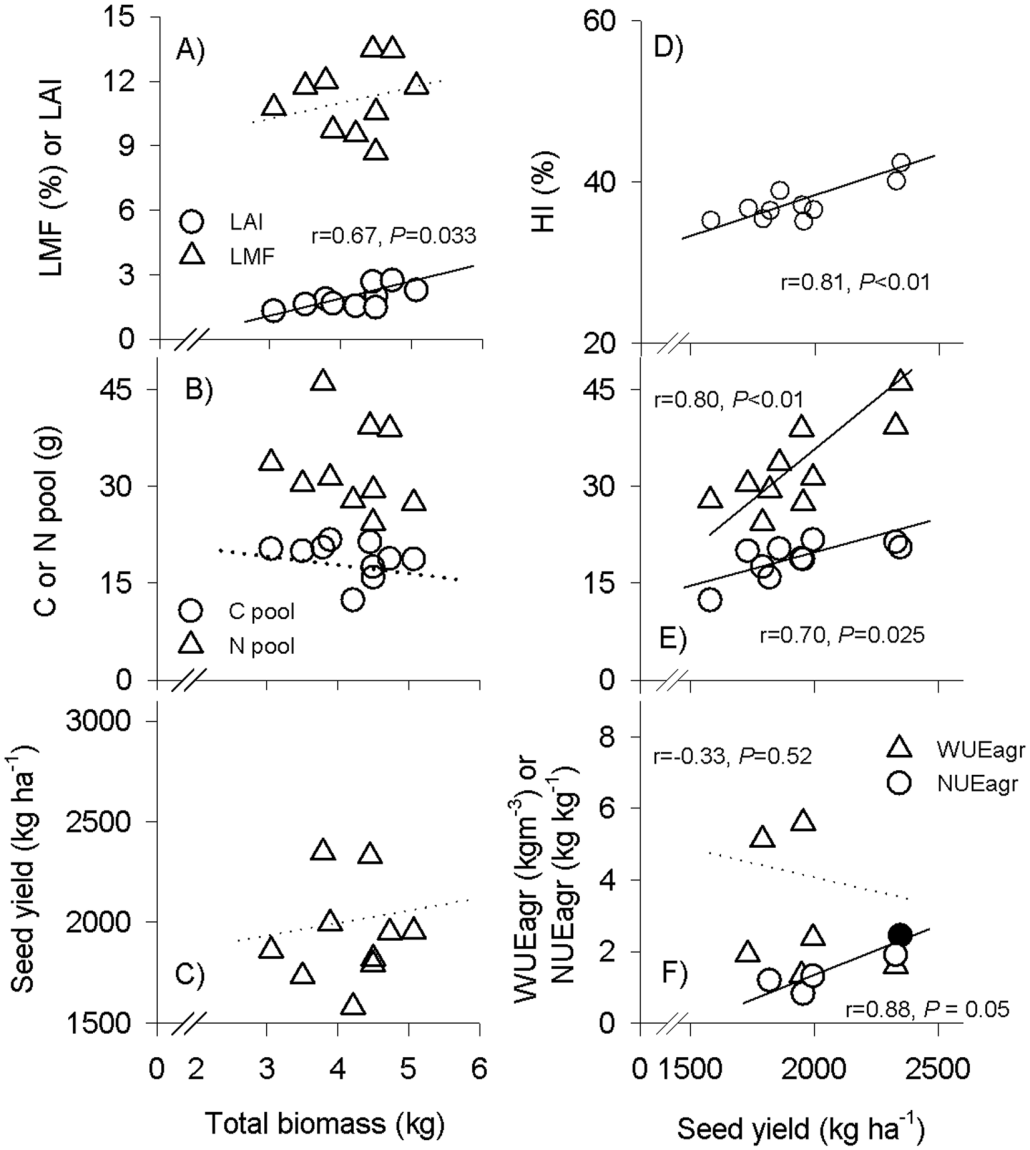
The relationship between plant biomass or total seed yield and morphological or physiological variables across different irrigation and fertilization treatments.

No significant interactions between irrigation and fertilizer were found for either the long-term water-use efficiency (indicated by leaf δ^13^C) or agronomic water-use efficiency (WUEagr = seed yield per water applied) (Fig. 4A,B). Leaf δ^13^C value was not influenced by fertilizer or irrigation (Fig. 4A). WUEagr was increased by fertilization, but decreased with the increase of irrigation amount (Fig. 4B). Across all irrigation treatments, agronomic nutrient-use efficiency (NUEagr = seed yield increase per fertilizer applied) was highest in APRI20 and FI, whereas it generally increased with increasing of irrigation amounts in DI regimes (Fig. 4C). WUEagr was negatively related with NUEagr in DI regimes (r = −0.92, *P* < 0.01). Total plant biomass was positively related to LAI across all treatments, but there was a negative trend between total biomass and soluble sugar pool in the stem (Fig. 5). Although not significantly related with total biomass, total seed yield was positively related to HI, total nitrogen pool of the vegetative organs, soluble sugar pool in stem, and NUEagr across all treatments (Fig. 5D,E,F), but there was a negative trend between total seed yield and WUEagr (Fig. 5).

## Discussion

### Seedlings were more sensitive to water deficit than large plants

The sensitivity of a plant’s growth stage to water deficit can be affected by many factors, including climatic conditions, crop species and cultivars, intensity and duration of water deficit, and agronomic management practices^6,27^. For example, under a Mediterranean climate, the most sensitive growth stage of wheat is at stem elongation and booting, followed by anthesis and grain filling^28^; whereas in North China Plains, wheat plants respond to water deficit more sensitively in the post-tillering stage than in the earlier stages^29^. Using the crop water production models, Igbadun *et al*.^30^ suggested that good yield of maize could be obtained with regular irrigation at the flowering stage, even if the irrigation is limited during the vegetative and seed-filling stages. Usually, water stress at the vegetative stage is more detrimental to biomass accumulation compared to that at the reproductive stage^8,15^. In line with this, RDI severely reduced total biomass in vegetative seedlings of *P. volubilis*, rather than that of large field-grown plants at the reproductive stage (Tables 1 and 2). The high sensitivity of seedling growth in response to RDI was attributed to the obvious reduction in leaf area (e.g. LAI) and photosynthetic rate. In response to RDI, the lower sensitivity of plant growth of large *P. volubilis* plants at the reproductive stage contrasted with annual oilseed crops, such as rape (*Brassica napus* L.), maize, and soybean^15,31,32^. Compared with FI, RDI increased root to shoot ratio (R/S) of the free-standing seedlings (Table 1), because water stress induced ABA accumulation is generally regarded as an inhibitor of shoot growth^4^. Consequently, the increased root mass fraction enhances the drought resistance of plants, so that the crop maybe better adapted to soil water deficit, providing benefits for water and nutrient uptake once FI resumes later in their life cycle^29^. Probably owing to their very large leaf and stem proportion but very small root proportion of a liana species^33^, no consistent effect on R/S ratio of the large field-grown *P. volubilis* plants was found in response to RDI (Table 2). Surprisingly, at the same amount of irrigation, an advantage of APRI over DI on plant growth was found in seedlings, rather than in large plants (Tables 1 and 2). Comparing APRI with DI in the field-grown grapevines receiving the same irrigation amount, the results also revealed only subtle differences in vegetative development, physiological response (e.g. stomatal behavior and the ABA contributions of root systems to leaf xylem ABA concentration), water use efficiency, and crop yield^14,34^. These controversial results between APRI and DI techniques may have occurred because the regulation of vegetative growth by RDI is complex and depends on depends on the crop growing-stage dependent differences in the long-transport hydraulic and chemical signals^4^ and total soil water availability^7^.

Representing the area through which water must diffuse to the leaves, the reduced SLA of both vegetative seedlings and large reproductive plants of *P. volubilis* with the decreasing amounts of irrigation contributed to conserving water and maintaining leaf function^1,29^. Gs in leaves of both seedlings and large plants of *P. volubilis* in the hot and dry season decreased, and thus reducing water loss through transpiration under drought conditions (Table 1, Fig. 1C). Meanwhile, the strong linear relationship between Pn and Gs indicated that stomatal closure due to water stress affected CO_2_ diffusion from the atmosphere to the site of carboxylation, resulting in Pn reduction^1^. However, although Pn, Gs and Tr in the hot and dry season decreased with decreasing amount of the irrigation, the effects of irrigation on leaf photosynthetic traits were only observed in the hot and dry season (Fig. 1); leaf physiological performance in the dry season can be fully recovered in the wet season^19^.

On a whole plant basis, nitrogen and mobile non-structural carbohydrates (mainly starch and soluble sugars) indicate a plant’s N and C supply status and reflect its capital for flushing, reproduction, and its buffering capacity with respect to abiotic stress^35^. Fertilization increased total nitrogen pool in the vegetative tissues and soluble sugar pool in stem (Fig. 2), indicating that fertilization increased plant C and N storages. The negative trend between C pools and plant growth (Fig. 5B) provided an evidence that the growth decline of *P. volubilis* plants under drought conditions depended on a factor such as reduction in turgor-driven cell expansion and/or constraint on phloem transport, rather than local C storages. It was also hypothesized that carbohydrates, as an active carbon sink, may be maintained prior to the growth of woody plants under drought conditions^36^. Furthermore, given that carbohydrates are products of photosynthates and substrates for tissue formation (i.e. growth) and maintenance processes (e.g. respiration), associations between changes in carbohydrate concentration and plant growth rate could help to explain whether source–sink dynamics influence the pattern of physiological acclimation of *P. volubilis* plants to a fluctuating environment^37,38^.

### Seed number was mainly responsible for the total seed oil yield

Irrigation and fertilization did not affect the pattern of fruit ripening of *P. volubilis* plants, which is in accordance with our previous studies^19,25,26,38^. This may be because a woody plant’s phenology with no requirement for vernalisation within a growing season is largely determined by its responses to temperature and photoperiod^39,40^. In line with our previous fertilization experiments^19,25^, both total seed and seed oil yield were increased by fertilization (Fig. 3C,D), but the magnitude of increase in total seed or seed oil yield by fertilization was similar among different irrigation regimes (no irrigation × fertilization interaction). However, the interactions of irrigation and fertilizer on the yield were found in other crops. For example, maize requires less N to achieve the maximum grain yield under the limited water supply condition when compared to the well-irrigated condition^22^. More N rate obviously increased the yield under moderate drought, but this might cause a yield reduction of coffee (*Coffea arabica*) plants under severe drought^41^. In oilseed crops, abiotic stress during the reproductive stage likely resulted in reductions in seed size and seed oil concentration, due to a decrease of assimilates available for fruit growth and to a higher competition for assimilates between sink tissues^24,37^. Seed oil concentration of oilseed crops in response to drought have produced various results, ranging from an increase to decreas^17,31,32^. Such variations can be explained by the fact that various soil water contents had different effects on metabolism, biosynthesis and accumulation of seed oils^42^. Neither irrigation nor fertilization significantly affected the mean seed size and mean seed oil concentration under different irrigation and fertilization treatments (Fig. 3A,B), which was consistent with our previous researches that seed size and seed oil concentration had a relatively high and constant heritability in *P. volubilis* plants in response to agricultural management practices such as water, fertilization and planting density^19,25,26^. Therefore, the increased seed (fruit) numbers per unit area was largely responsible for the influences of irrigation and fertilization on the total seed oil yield of *P. volubilis* plants. The highest values of total seed and seed oil yield of *P. volubilis* plants were found in APRI20 and FI combined with fertilization (Fig. 3C,E), indicating DI decreased the yield. The high values of total seed and seed oil yield in APRI20 suggested that small amounts of water applied in the dry season could elicit substantial yield gains, which were also found in olive tree and wine grapes (*Vitis vinifera* L.)^15^. As non-additive effect of irrigation on total seed yield was found between the two years; the higher total seed yield in 2016 than in 2015 can probably be attributed to the larger plant size. Moreover, the mature stage of *P. volubilis* plants coincided with the dry season in our studied region (Table 3), and natural drought decreased the number of flowers and increased the abortion of fruitlets^19^. Thus, a proper combination of irrigation and fertilizer can achieve the optimal coupling effect and obtain a higher yield. This might be associated with the improvement of the reproductive development of *P. volubilis* plants under full irrigation or APRI technique combined with higher fertilization rates.

Seed production in the *P. volubilis* plant, a wind-dispersed species with well-developed reproductive organs^19,23^, depends mainly on the availability of current photo-assimilates and storage resources (i.e., carbohydrates and N)^35,43^. The positive relationships between total seed or seed oil yield and C or N pools across different irrigation regimes (Fig. 5E) indicated that C and N shortages are essential for the yield formation, especially when current photo-assimilates were limited during the reproductive stage in the dry season (Fig. 1). Carbohydrate availability can influence yield by adjusting the fruit number to the metabolite supply of the plant through the premature abscission of flowers and developing fruits^35,38^. However, it has also been reported that the status of carbohydrates is not a yield determinant of large tree crops, such as cacao^44^ and olive tree^45^. The significance of N for boosting *P. volubilis* productivity agreed with our previous report^25^. The increased seed yield was not accompanied by an increased plant biomass across different irrigation and fertilization treatments (Fig. 5D), contrasting with our previous results of *P. volubilis* plants in response to light intensity, fertilization and planting density^19,23,25^. RDI are known to enhance the source-sink relationship (increases in C and N shortages and harvest index; Table 2 and Fig. 2) and to stimulate the translocation of photo-assimilates, thereby helping in effective flower formation, seed development and ultimately enhancing the productivity of crops^7,37^.

### APRI was successful in reducing water and fertilizers used

Physiologically, WUEi describes the intrinsic trade-off between carbon fixation and water loss, because water evaporates from the interstitial tissues of leaves whenever stomata open to uptake CO_2_ acquisition for photosynthesis^1^. The increased WUEi of both seedlings and large *P. volubilis* plants in the hot and dry season with decreasing amounts of the irrigation arise from the fact that Gs was reduced more than Pn. However, stomatal closure contributes to increasing WUEi on the one hand, while decreasing PNUE on the other hand, resulting in a trade-off between both traits, especially during drought^1,46,47^. The negative relationships between WUEi and PNUE of both seedlings and large *P. volubilis* plants in the hot and dry season were consistent with this concept at the leaf scale. WUEi is time- and labor-intensive to measure for large numbers of plants, and it also fluctuates with normal environmental variation in the field. Therefore, proxy traits for WUE, such as carbon isotope discrimination (δ^13^C) and yield accumulation (e.g., agricultural water use efficiency, WUEagr), can provide more integrative long-term WUE trends. The positive linear relationships between δ^13^C and WUEi have been reported in some plant species^46,48^, but not in our studied species (Fig. 4A). Representing a long-term assimilation of weighted integration of Ci/Ca, leaf δ^13^C cannot be always be simply related to WUEi because of differences in the respective time of integration^48^ or variable mesophyll diffusion conductance^46^. Our hypothesis was that such representativeness could be improved by choosing an adequate sample and sampling time. The relationship between δ^13^C and WUE in grapevines also shows great variations among different experiments, thus limiting its interest as an indicator of water status or WUE^48^. WUEagr, but leaf δ^13^C value, was affected by fertilizer or irrigation (Fig. 4A,B). Regardless of irrigation treatments, WUEagr increased by fertilization with the highest values found in APRI20 and FI; whereas it increased with decreasing amounts of the irrigation in DI regimes. Such a water-saving phenomenon of using RDI to reduce the amount of irrigation and increase WUE was also widely reported in herbal^9,31,49^ and woody crops^8,14,29^ with little or no yield penalty in most cases. The negative trend between total seed yield and WUEagr in DI regimes (Fig. 5F) indicated that maximum yield and maximum water productivity (yield divided by irrigated water applied) are not always compatible goals^15,26^.

In rice and maize plantation systems, adapting water management may help to mitigate N loss, hence increasing yield and NUEagr^2,50^. This was also the case for the *P. volubilis* plant, as NUEagr was positively related to total seed yield across different irrigation regimes (Fig. 5F). As the oil production in *P. volubilis* plants required high levels of fertilizer^25^, the maximum NUEagr is expected when water inputs are close to the crop’s water demand or when a suitable water-saving irrigation technique applied (e.g., APRI in this study), whereas sub-optimal DI leads to decreased NUEagr. The fact that APRI20 achieved a higher seed yield, WUEagr and NUEagr simultaneously of *P. volubilis* plants may be due to the restrained growth redundancy and improved canopy structure (lower total biomass but higher LAI and HI; Table 2), thus reducing the water and N used in the production of vegetative tissues and the water used for transpiration from redundant leaf areas. It was also reported that APRI improved the ability of plants to acquire nutrients from the soil because soil drying and wetting cycles stimulated the mineralization of soil organic N and microbial activity, thereby increasing NUEagr^21,51^. On the other hand, the well-known trade-off between water-use efficiency and nitrogen-use efficiency was also confirmed by the negative relationship between WUEagr and NUEagr at the field scale. But maintaining a high yield and NUEagr at the cost of WUEagr is recommended for *P. volubilis* plantation in our studied water-rich areas.

## Conclusion

In response to water deficit, the high sensitivity of seedling growth was attributed to the great reduction in leaf area and photosynthetic rate, whereas the growth of the large field-grown *P. volubilis* plants at the reproductive stage had a low sensitivity. The fast growth in vegetative seedlings of *P. volubilis* plants can be achieved by irrigation applied to meet full evapotranspiration at the cost of whole-plant water-use efficiency, because both APRI and DI significantly reduced their normal growth. Compared with DI at the same amount of irrigation, APRI was more efficient in improving the whole-plant water-use efficiency of the vegetative seedlings and the irrigated water-use efficiency of large reproductive plants. The total seed oil yield of *P. volubilis* plants was largely determined by the seed (fruit) numbers per unit area, rather than by seed size or seed oil concentration, across irrigation and fertilization treatments. The magnitude of increase in total seed and seed oil yield by fertilization was similar among different irrigation regimes, as no interactions of irrigation × fertilization were found. Carbon storage may be an active process, occurring at the expense of growth, whereas C and N shortages are essential for the yield formation under drought. The highest total seed yield and total seed oil yield were obtained in APRI20 and full irrigation when combined with fertilization. Compared with full irrigation, APRI20 had similar total seed oil yield and agronomic nutrient-use efficiency_,_ but reduced the irrigated water greatly. APRI with soil drying-rewetting cycles applied in the dry season was successful in increasing seed oil yield and reducing water and fertilizers used, even in a tropical humid monsoon area. Additional studies under the agricultural management practices with various levels of APRI and fertilizer in different plantation systems would be supportive.

## Materials and Methods

### Study site

**Figure 6:**
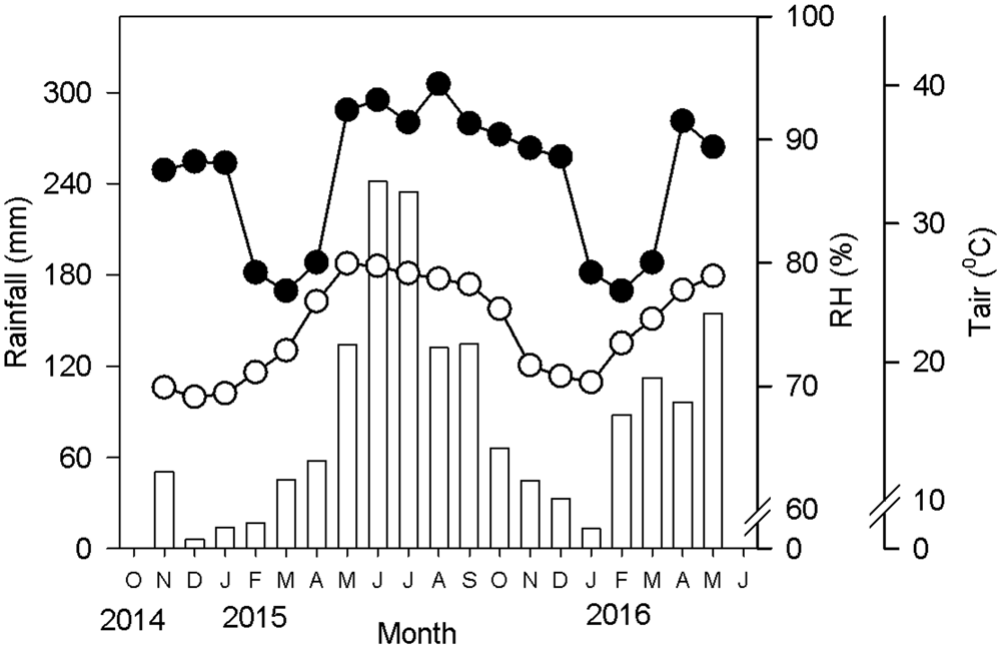
Seasonal changes in monthly precipitation (bars), mean air temperatures (○) and relative humidity (RH; ●) in the experiment conducted during 2014–2016.

Two separate experiments were conducted at the Xishuangbanna Tropical Botanical Garden (21°56′N, 101°15′E, altitude 560 m) in Xishuangbanna, SW China. The climate at Xishuangbanna is dominated by the southwest monsoon, which has two distinct seasons (a wet season from May to October, and a dry season from November to April). The average annual temperature is 22.9 °C, and the mean annual precipitation is 1,500 mm, of which approximately 85% occurs in the wet season; the relative humidity is very high throughout the years (>74%) (Fig. 6). According to the mean monthly air temperature, the dry season can be divided into the cool and dry season (November to January) and the hot and dry season (February to April)^27^.

### Experiment 1

#### seedling experiment in the greenhouse

The seedling experiment was carried out in the greenhouse from November, 2013 to January, 2014. The average temperature inside the greenhouse was about 21 °C. Mature seeds of *P. volubilis* were nursed in the sandy soil. The growth-uniform young seedlings of *P. volubilis*, in height of about 20 cm, were transplanted to pots (trapeziform cylinder in shape, inner diameter 26 cm, height 23 cm; 7 kg soil); one plant per pot. The pots with the stem stump in all treatments were covered with plastic film to reduce soil evaporation. The physical and chemical properties of the soil were measured according to the method described by Dewis and Freitas (1970)^52^ and are as follows: organic matter 18.39 g kg^−1^, available nitrogen 110 mg kg^−1^, available phosphorus 5.98 mg kg^−1^, available potassium 98 mg kg^−1^. The bulk soil density is approximately 1.15 g cm^−3^.

All seedlings were well irrigated to field water capacity during the first 2 weeks until fully established. Then, the initial dry biomass (W_1_) of seedlings with 5 individuals was measured before the start of the irrigation treatments. A total of two factors were designed, i.e., irrigation strategy and irrigation amount. The irrigation strategies included conventional deficit irrigation (DI, the roots were irrigated evenly) and alternative partial root-zone irrigation (APRI, the roots in the two parts were irrigated alternatively). Because serious drought caused the death of the seedlings, the irrigation amount covered three relatively high levels, that is, full irrigation (FI, 100% evapotranspiration (ET); irrigated evenly), 75% and 50% of ET (DI or APRI). The FI plants were fully irrigated every 2 days in the late afternoon according to weight loss of each pot, and the reduced soil water content was maintained at pot water holding capacity. The DI and APRI plants received a percentage of the average water volume of the FI plants at each irrigation event during the treatment period. A total of five treatments were designed with 20 pots for every treatment; each pot was totally irrigated 26 times during the 12-weeks experimental period. In the APRI treatment, a single plant was placed in a split-root pot. The compartments within each pot was equally separated by a wooden board (0.70 × 0.20 cm) and was lined with black polyethylene (1.2 mm thickness) to avoid movement of water from one compartment to the other; each side of the container was filled with 3.5 kg soil. Deep leakage did not occur because of shallow depth of wetted-soil of irrigation in this experiment. Deep leakage did not occur because of the shallow depth of the wetted-soil of used for irrigation.

#### Measurements

At the end of January 2014, leaf gas exchange parameters (Pn, net photosynthetic rate; Tr, transpiration rate; and Gs, stomatal conductance) of fully expanded mature leaves were measured with a portable photosynthesis measurement system (Li-6400XT, Li-Cor, Lincoln, NE, USA) under saturated light intensity (1800 μmol m^−2^s^−1^) during 8:30–11:00 on sunny days. The instantaneous water-use efficiency (WUEi, Pn/Tr) was calculated. Five to six plants were harvested randomly for every treatment. The fresh blades were scanned with a CanoScan 4400 F scanner, and their leaf area indexes were calculated using ImagJ software. The seedlings (vegetative stage; free standing) were divided into roots, stems and leaves, which were dried at 70 °C to constant weights (W_2_). Leaf nitrogen (N) concentration was determined by micro Kjeldahl digestion. Photosynthetic N utilization efficiency (PNUE, Pn/N) and whole plant water-use efficiency [WUEwp = (W_2_ -W_1_)/amount of irrigated water] were calculated. Specific leaf area (SLA; i.e., area of the leaf in cm^2^ g^−1^ DW) and leaf area index (LAI) were also calculated.

The field experiments were arranged in a split-plot design with randomized complete blocks and three to four replications in a 2 × 40 m sized plot. Fertilization rates were assigned to the main plots and consisted of 200 kg ha^−1^ and 0 kg ha^−1^ of a 1:1:1 (w/w/w) mix of N:P_2_O_5_:K_2_O spread in an approximately 1.0 m-wide zone in June in 2014 and 2015, respectively, according to previous research (Yang *et al*.^25^). Irrigation was assigned to the sub-plots, including rainfed (control) and four levels of irrigation regimes [APRI20 and DI20, DI50, and full irrigation; with irrigation amounts of 20, 50 and 100% crop evapotranspiration (ETc), respectively] from early December to late April in the dry season; the plots were irrigated once every second week. Irrigation was built between blocks, and the amount of irrigation water was monitored with flow meters (LXSG-50 Flow meter, Shanghai Water Meter Manufacturing Factory, Shanghai, China) installed in the irrigation pipelines. Each sub-plot was irrigated independently. Two pipelines with emitters were joined at both sides of the trunk and placed underneath each row. In each pipeline for the APRI treatment, there were dry and wet root zones with alternative irrigation at each side within each row. In the full irrigation and DI treatments, irrigation water was supplied simultaneously to both sides of the root system. Crop evapotranspiration (ETc = ET_0_ × K_c_) was estimated using crop coefficients (K_c_) based on those proposed by the FAO and reference evapotranspiration (ET_0_) values, and were calculated by the Penman-Monteith-FAO method^53^ and using the daily climatic data collected in the Xishuangbanna Station for Tropical Rain Forest Ecosystem Studies (XSTRES) nearby. Crop coefficients (K_c_) of the field-grown *P. volubilis* plants in this study was estimated as 1.0 with the reference to tropical fruit trees and grapevine.

#### Measurements

Leaf gas exchange parameters were measured under light-saturating irradiance (photosynthetic photon flux density = 1,800 μmol m^−2^s^−1^) and ambient CO_2_ concentration on recently matured, sun canopy leaves, using a portable infrared gas analyzer in open system mode (*LI-6400XT*) in January, April and July, respectively, in 2015; subsample of leaves was also collected for the measurement of N concentration. Mature fruit from all *P. volubilis* plants were harvested five-times by hand for each sub-plot throughout the period of fruit ripening. The total dry mass (DM) of fruit per plot was measured at each harvest; sub-samples of harvested fruit were peeled, and the dry weight (size) of seeds was recorded. Seed oil concentrations were determined by the minispec mq-one Seed Analyzer (*Bruker Optik GmbH*, Germany); the total seed oil yield (kg ha^−1^) throughout the growing season was then calculated by the sum of the values from each harvest.

Four to five plants were harvested from each treatment in late April 2015. The plants were separated into leaves, stems, roots, and fruit [both green (less than 2%) and mature]; and were dried to a constant mass and weighed. Then, the biomass fraction of each component was calculated. For the calculation of total plant biomass and fruit mass fraction (i.e., harvest index), the value of total fruit yield throughout the year was used. Sub-samples of leaves were scanned to determine leaf area; SLA and LAI were then calculated. N concentration of vegetative tissues (i.e. leaf, stem and root) was determined by micro Kjeldahl digestion. The dried stem tissue was also analyzed for the total soluble sugar (glucose, fructose, and sucrose) concentration, following UV spectrophotometry methods modified from Dubois *et al*.^54^. Total nitrogen pool of the vegetative tissues was calculated with the sum of the N concentration multiplied by the dry weight of each tissue; soluble sugar pool in stem was calculated by the N concentration in stem multiplied by the dry weight of stem. Agronomic water-use efficiency (WUEagr) was calculated as kg seed yield per water applied as irrigation, that is, IWUE = seed yield/amount of irrigated water. Agronomic nutrient-use efficiency (NUEagr) was calculated as: NUEagr = (total seed yield in fertilized plot – total seed yield in non-fertilized plot)/fertilizer used in fertilized plot.

Dry subsamples of leaf δ^13^C values were determined by an isotope ratio mass spectrometer (MAT DELTA^PLUS^XL, Thermo Finnigan, USA, analytical precision was about 0.1%). The results were then expressed as δ^13^ C values to characterize leaf long-term water use efficiency (WUE), using a formula as:

1 $${\delta }^{{\rm{13}}}{\rm{C}}( \% )=[({{\rm{R}}}_{{\rm{sample}}}-{{\rm{R}}}_{{\rm{standard}}}){/R}_{{\rm{standard}}}]\times {\rm{1000}}$$

where R_sample_ and R_standard_ are the isotope ratio (^13^C/^12^C) of samples and of the international standard Vienna-Pee Dee Belemnite (VPDB), respectively.

### Statistical analysis

Differences in the values of each variable of seedlings to the irrigation treatments were tested by one-way ANOVA, followed by a Tukey HSD post hoc test. For the variables of the field-grown plants, data were analysed with a two-way ANOVA, with irrigation (I) and fertilization (F) as the main fixed factors, plus an I × F interaction term. Data were checked for normality and homogeneity of variances, and a log_10_ or square-root transformation was applied when necessary to satisfy the assumptions of ANOVA. Correlations amongst traits were analyzed with a Pearson’s correlation; all reported correlations were significant at an alpha level of *P* < 0.05. All statistical analyses were conducted using SPSS version 21.0 (SPSS, Chicago, IL, USA).
